# Spontaneous apoptosis of cells in therapeutic stem cell preparation exert immunomodulatory effects through release of phosphatidylserine

**DOI:** 10.1038/s41392-021-00688-z

**Published:** 2021-07-14

**Authors:** Xuemei He, Weiqi Hong, Jingyun Yang, Hong Lei, Tianqi Lu, Cai He, Zhenfei Bi, Xiangyu Pan, Yu Liu, Lunzhi Dai, Wei Wang, Canhua Huang, Hongxin Deng, Xiawei Wei

**Affiliations:** 1 Laboratory of Aging Research and Cancer Drug Target, State Key Laboratory of Biotherapy and Cancer Center, National Clinical Research Center for Geriatrics, West China Hospital, Sichuan University, Chengdu, Sichuan People’s Republic of China; 2 Experimental Medicine Center, the Affiliated Hospital of Southwest Medical University, Luzhou, Sichuan People’s Republic of China

**Keywords:** Immunological disorders, Inflammation

## Abstract

Mesenchymal stem cell (MSC)-mediated immunomodulation has been harnessed for the treatment of human diseases, but its underlying mechanism has not been fully understood. Dead cells, including apoptotic cells have immunomodulatory properties. It has been repeatedly reported that the proportion of nonviable MSCs in a MSC therapeutic preparation varied from 5~50% in the ongoing clinical trials. It is conceivable that the nonviable cells in a MSC therapeutic preparation may play a role in the therapeutic effects of MSCs. We found that the MSC therapeutic preparation in the present study had about 5% dead MSCs (DMSCs), characterized by apoptotic cells. Namely, 1 × 10^6^ MSCs in the preparation contained about 5 × 10^4^ DMSCs. We found that the treatment with even 5 × 10^4^ DMSCs alone had the equal therapeutic effects as with 1 × 10^6^ MSCs. This protective effect of the dead MSCs alone was confirmed in four mouse models, including concanavalin A (ConA)- and carbon tetrachloride (CCl_4_)-induced acute liver injury, LPS-induced lung injury and spinal cord injury. We also found that the infused MSCs died by apoptosis in vivo. Furthermore, the therapeutic effect was attributed to the elevated level of phosphatidylserine (PS) upon the injection of MSCs or DMSCs. The direct administration of PS liposomes (PSLs) mimic apoptotic cell fragments also exerted the protective effects as MSCs and DMSCs. The Mer tyrosine kinase (MerTK) deficiency or the knockout of chemokine receptor C–C motif chemokine receptor 2 (CCR2) reversed these protective effects of MSCs or DMSCs. These results revealed that DMSCs alone in the therapeutic stem cell preparation or the apoptotic cells induced in vivo may exert the same immunomodulatory property as the “living MSCs preparation” through releasing PS, which was further recognized by MerTK and participated in modulating immune cells.

## Introduction

Mesenchymal stem cell (MSC)-based therapy has been recognized as a promising option for the life-threatening diseases such as acute myocardial injury, liver failure, lung injury, stroke, hematopoietic disorders, etc.^1–6^ MSCs derived from the bone marrow, umbilical cord, or adipose tissue have the potential to differentiate into various cell lineages, thus replacing the damaged tissues,^7^ which makes MSCs candidates for cell-based therapeutic strategies for certain diseases. Studies reported that MSCs possess immunomodulatory potential in both innate immunity and adaptive immunity, and MSCs regulate the functions of various immune cells through cell-to-cell contact and paracrine activity.^8,9^ However, the molecular mechanisms of MSC-mediated immunomodulation remain unclear.

Many previous studies hold the idea that implanted MSCs could reach to injured site and further regulate the immune-microenvironment. It is reported that the therapeutic potentials of MSCs mainly attribute to the replacement of the damaged tissues by differentiating into various cell lineages and the secretion of regulating factors, including immunomodulatory factors, angiogenic factors, anti‑apoptotic factors, and antioxidative factors,^10,11^ which are based on the viability of MSCs. However, recent studies have found that MSCs are short-lived after transplantation into recipients.^12,13^ For instance, only a few surviving MSCs have been observed in injured brain, liver, or spinal cord.^6,14,15^ Moreover, during the preparation process of the MSC-based cell therapies, it is hard to avoid cell deaths. The proportion of nonviable MSCs in an injected preparation varied approximately from 5~50% during the reported clinical trials (Supplementary Table S1), which makes it unclear whether the nonviable cells also play a role in the therapeutic effect of MSCs.

The immunomodulatory effects of apoptotic cells have been reported. The phagocytosis of apoptotic cells by monocytes/macrophages blocks the production of inflammatory cytokines, regulates inflammation-related signaling such as nuclear factor-κB (NF-κB), liver X receptor (LXR), and phosphatidyl inositol 3-kinase (PI3K), and promotes the release of anti-inflammatory factors including transforming growth factor-β (TGF-β), prostaglandin E2 (PGE2), and hepatocyte growth factor (HGF).^16–19^ Exposure of phosphatidylserine (PS) in the outer leaflet of the plasma membrane is the best-characterized feature of apoptotic cells.^20^ PS eversion not only provides an eat-me signal but also functions as a dominant immunosuppressive signal, which promotes tolerance and prevents local and systemic immune activation.^21,22^ In the current study, we hypothesized that nonviral MSCs might also have functional roles in the therapeutic effects of the MSCs through regulating the local immune-microenvironment by releasing/resenting apoptotic factors. Therefore, in this study, we administered living MSCs and dead MSCs (DMSCs) to mice of four injury models, to evaluate the therapeutic effects, and investigated the underlying mechanisms for immunomodulating capacity of DMSCs.

## Results

### Characterization of bone marrow-derived MSCs

**Fig. 1:**
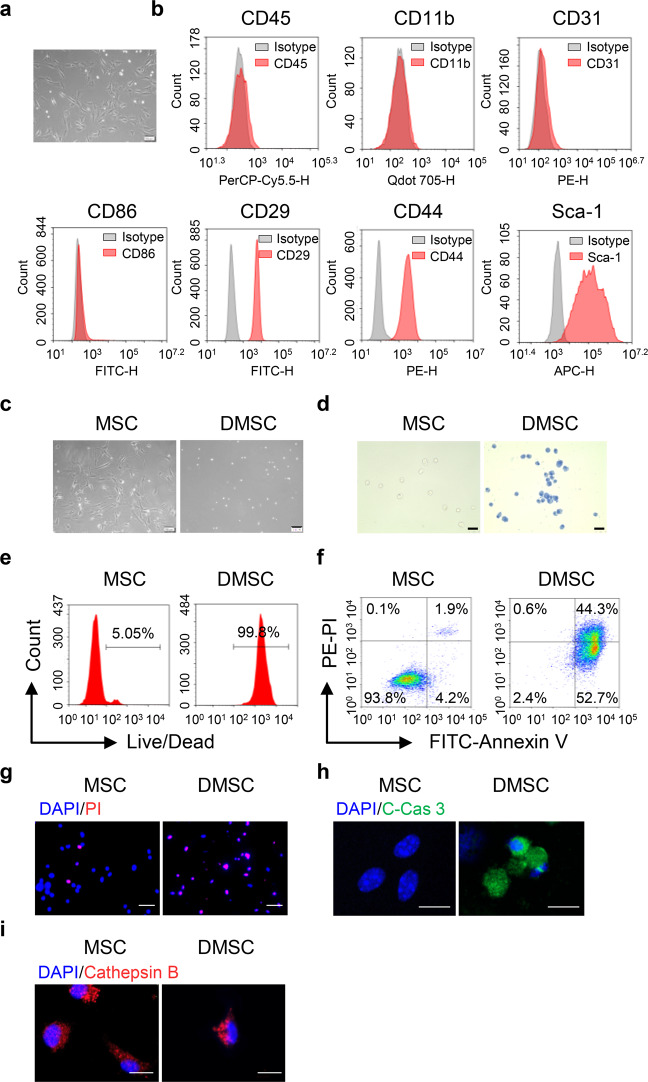
Culture and identification of MSCs and DMSCs. **a** The morphology of MSCs. Scale bar represents 100 μm. **b** MSCs were identified as CD45^−^CD11b^−^CD31^−^CD86^−^CD29^+^CD44^+^Sca-1^+^. **c** Morphology of living MSCs and DMSCs. Scale bar represents 100 μm. **d** Identification of living and dead MSCs by Trypan blue. Scale bar represents 100 μm. **e** Living and dead MSCs were stained using LIVE/DEAD^TM^ Near-IR Dead Cell Stain Kit and were analyzed by flow cytometry. **f** The apoptosis of MSCs and DMSCs detected by flow cytometry. **g** The PI staining of MSCs and DMSCs. Scale bar represents 50 μm. **h**, **i** The expression of cleaved caspase 3 (**h**) and Cathepsin B (**i**) in MSCs and DMSCs. Scale bar represents 10 μm. C-Cas 3, cleaved caspase 3

MSCs were isolated from the bone marrow of 2- to 3-week-old mice as described previously.^23^ The isolated MSCs exhibited long-spindle shape (Fig. 1a) and were positive for CD29, CD44, and Sca-1 but negative for CD45, CD11b, CD86, and CD31 (Fig. 1b), which is in accordance with previous reports.^24–26^ Spontaneous cell deaths occur in the isolated MSCs. To distinguish DMSCs from MSCs, the living MSCs were adherent, whereas DMSCs were round and suspended in the medium (Fig. 1c). In addition, the living MSCs and DMSCs could be recognized by Trypan blue staining (Fig. 1d). In most cases, the MSCs are likely to contain ~5% DMSCs through the standard isolation and culture process, which was confirmed by flow cytometry (Fig. 1e). We further found that the DMSCs were Annexin V and cleaved caspase 3 positive (Fig. 1f–h). Diffusion of cytoplasmic Cathepsin B was considered as a marker of necrosis for the loss of lysosomal membrane integrity. We observed that Cathepsin B in MSCs and DMSCs were not diffused (Fig. 1i). These results indicated that DMSCs were apoptotic rather than necrotic cells.

### DMSCs attenuate tissue damage in four injury models

To determine the hepatoprotective effects of DMSCs alone in an acute liver injury (ALI) model, we induced ALI in mice by injecting concanavalin A (ConA) or carbon tetrachloride (CCl_4_).

**Fig. 2:**
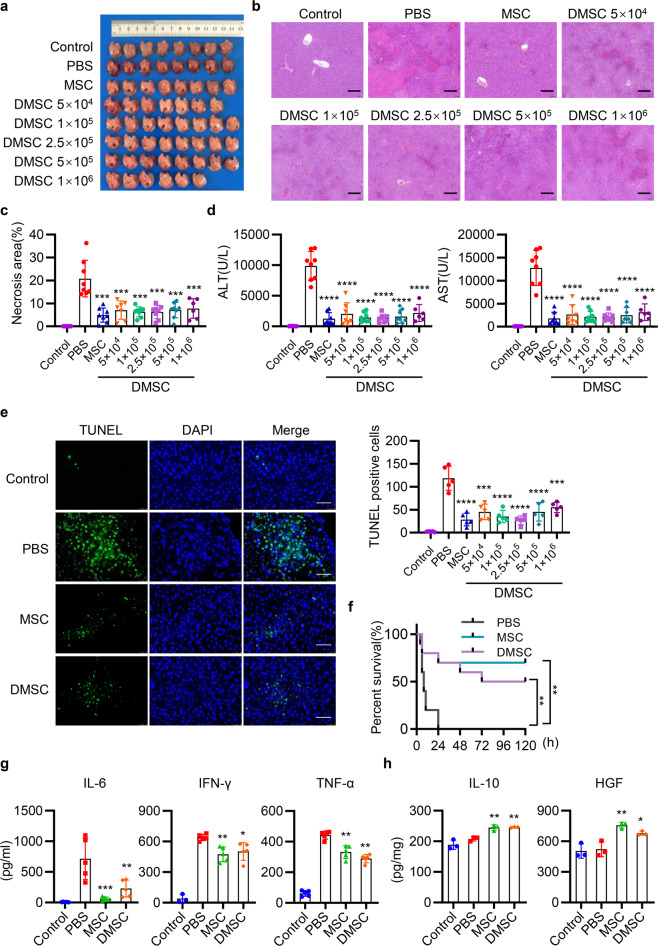
MSCs and DMSCs attenuate ConA-induced liver injury in mice. **a** Mice were intravenously injected with 12 mg/kg ConA, followed by intravenous injection with PBS, 1 × 10^6^ MSCs (containing 5 × 10^4^ DMSCs) or 5 × 10^4^ to 5 × 10^6^ DMSCs. Twelve hours after administration of ConA, mice were killed and representative macroscopic images of livers were shown. *n* = 6~8. **b** Representative images of liver histopathology with H&E staining. Scale bar represents 200 μm. **c**–**e** Quantitative analysis of necrotic area (**c**), serum ALT and AST levels (**d**), and TUNEL-positive cells (**e**, scale bar represents 50 μm) in mice with PBS, MSCs, or DMSCs treatment after ConA injection. *n* = 6~8 in **c**, **d** and *n* = 5 in **e**. **f** Survival of 25 mg/kg ConA-injected mice treated with PBS, MSCs, and DMSCs. *n* = 10. **g**, **h** The levels of IL-6, IFN-γ, and TNF-α in serum (**g**), and IL-10 and HGF in hepatic tissues (**h**) were determined by ELISA in each group. *n* = 3~5 in each group. Data are represented as mean ± SEM. ANOVA with Dunnett’s multiple comparison test was performed. Statistical significance is indicated by *p < 0.05, **p < 0.01, ***p < 0.001, ****p < 0.0001, compared with PBS group

To investigate the possible role of the apoptotic cells in our MSC therapeutic preparation for the immunomodulatory effect, we calculated the percentage of apoptotic cells by the flow cytometry and found that there were about 5% apoptotic cells in our MSC therapeutic preparation. Namely, 1 × 10^6^ MSCs contained 5 × 10^4^ DMSCs. Therefore, we investigated whether 5 × 10^4^ DMSCs or more DMSCs have similar therapeutic effects as 1 × 10^6^ MSCs. Mice were injected with ConA, followed by intravenous injection with 1 × 10^6^ MSCs (containing 5 × 10^4^ DMSCs) or 5 × 10^4^ to 5 × 10^6^ DMSCs alone. It is interesting to find that treatment with the DMSCs alone, even with the equal number of DMSCs (5 × 10^4^) is as effective as 1 × 10^6^ MSCs (containing 5 × 10^4^ DMSCs) in improving the structure of hepatic lobule, decreasing the range of liver necrotic area, reducing the levels of serum alanine aminotransferase (ALT), aspartate aminotransferase (AST), and number of apoptotic hepatocytes (Fig. 2a–e). Moreover, DMSCs alone, such as MSCs, promoted the mice survival compared with phosphate buffer solution (PBS) group (Fig. 2f). In addition, DMSCs reduced the levels of interleukin 6 (IL-6), interferon-γ (IFN-γ), and tumor necrosis factor-α (TNF-α) in serum and increased IL-10 and HGF levels in hepatic tissues (Fig. 2g, h). Taken together, the above results showed that DMSCs, even a small fraction containing in our MSC therapeutic preparation, displayed equal hepatoprotective properties as MSCs in alleviating ConA-induced liver injury.

**Fig. 3:**
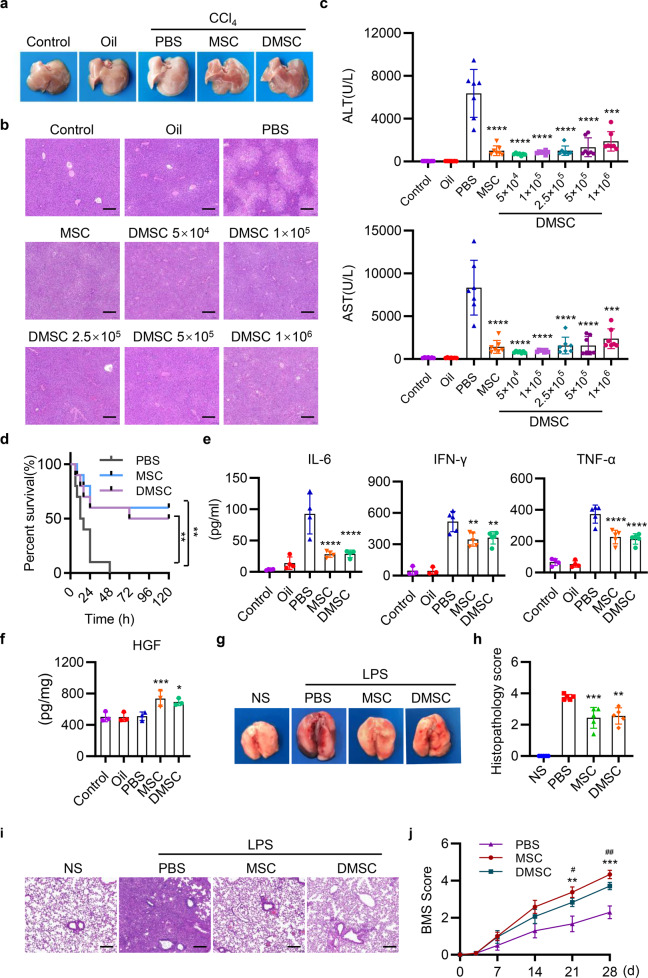
MSCs and DMSCs attenuate tissue injury in mice. **a** Mice were intraperitoneally injected with 0.5 ml/kg CCl_4_, followed by intravenous injection with PBS, MSCs, or DMSCs. Representative macroscopic images of livers 24 h after CCl_4_ injection. **b** Representative images of liver histopathology with H&E staining 24 h after CCl_4_ administration. Scale bar represents 200 μm. **c** Quantification of serum AST and ALT after CCl_4_ injection in each group. *n* = 7 in each group. **d** Survival 5 ml/kg CCl_4_-injected mice treated with PBS, MSCs, and DMSCs. *n* = 10. **e**, **f** The levels of IL-6, IFN-γ, TNF-α in serum (**e**) and HGF in hepatic tissues (**f**) were determined by ELISA in each group. *n* = 3~5 in each group. **g** Mice were intratracheally instilled with 5 mg/kg LPS, followed by intravenous injection with PBS, MSCs, or DMSCs. The representative macroscopic images of the lungs 72 h after LPS administration were shown. **h**, **i** Representative images of lung histopathology with H&E staining (**i**) and histopathology score (**h**) 72 h after LPS administration. Scale bar represents 200 μm. **j** MSCs and DMSCs improved the behavior of mice in spinal cord injury model. Data are represented as mean ± SEM. *p < 0.05, **p < 0.01, ***p < 0.001, ****p < 0.0001, compared with PBS group in **c**–**f**, **h**. **p < 0.01, ***p < 0.001, MSC compared with PBS group; ^#^*p* < 0.05, ^##^*p* < 0.01, DMSC compared with PBS group in **j**

Hepatoprotective effects were also observed in CCl_4_-induced liver injury by using only 5 × 10^4^ DMSCs or more DMSCs. The DMSCs alone alleviated pathological damages, reduced levels of serum AST, ALT, and proinflammatory cytokine, and increased levels of HGF (Fig. 3a–c, e, f). Notably, MSCs and DMSCs injection also improved the survival of mice in CCl_4_-induced ALI (Fig. 3d). Besides, in lipopolysaccharide (LPS)-induced lung injury model, we observed that the instillation of LPS-induced inflammatory infiltrates, interalveolar septal thickening, and interstitial edema. Injection of equal number of DMSCs (5 × 10^4^) in our DMSC-containing MSCs preparation (1 × 10^6^ MSCs, with 5 × 10^4^ DMSCs) attenuated lung damages and reduced the histopathology score of lung sections (Fig. 3g–i). In addition, similar protective role of the DMSCs was observed in mice with spinal cord injury (SCI) for increasing the Basso mouse scale (BMS) score of SCI mice (Fig. 3j).

### Transplanted MSCs undergo apoptosis and release phospholipids in vivo

**Fig. 4:**
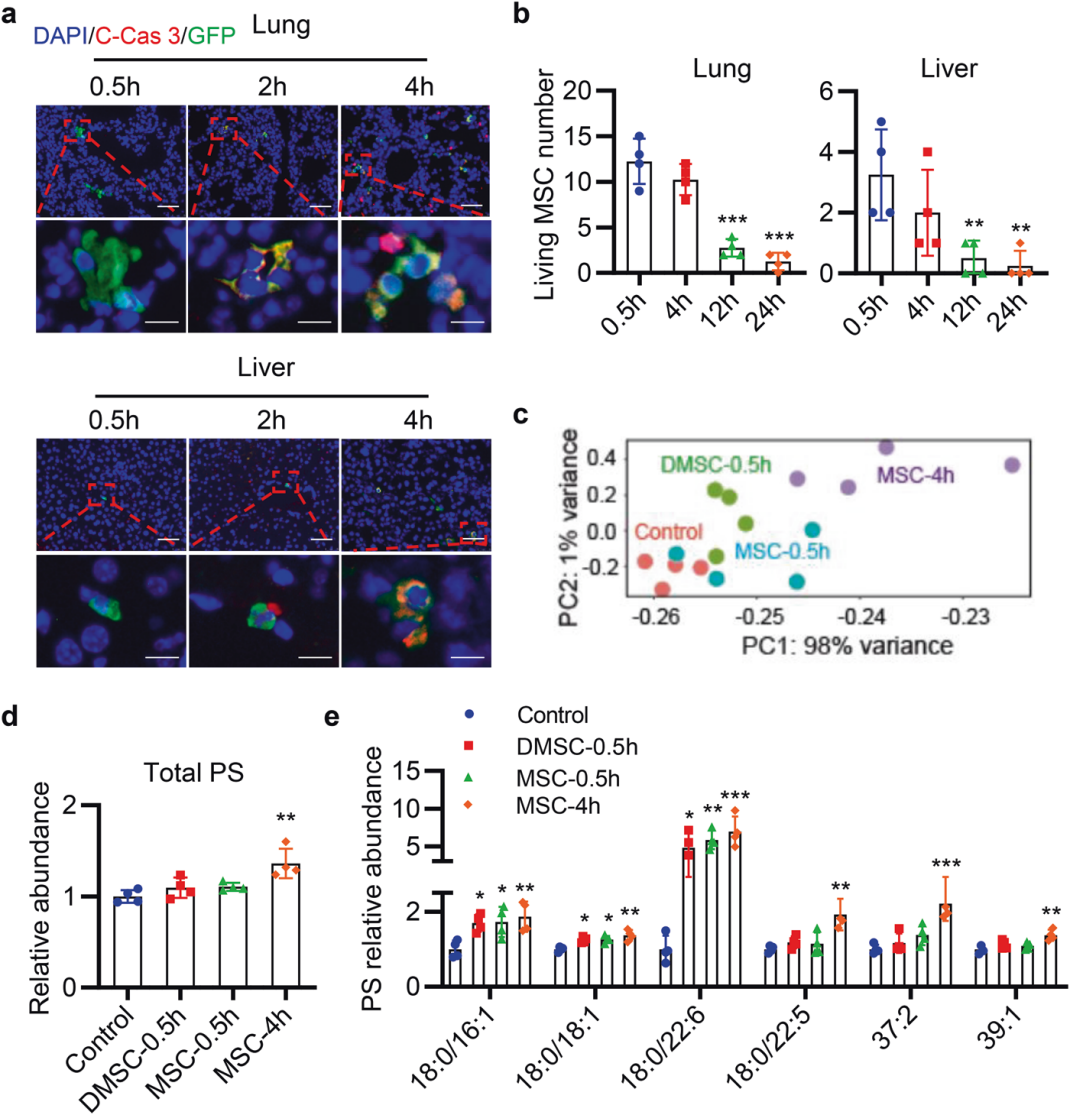
Transplanted MSCs undergo apoptosis within few hours and release phospholipid in vivo. **a** Here, 1 × 10^6^ GFP-MSCs were injected into mice by i.v. and the lung and liver tissues were taken for frozen section and stained for C-Cas 3 (red) and DAPI (blue) at 0.5, 2, and 4 h. Scale bar represents 50 μm (upper row) or 10 μm (lower panel). **b** Quantification of living MSC number in the lung and liver after GFP-MSCs injection. **p < 0.01, ***p < 0.001, compared with the 0.5 h group. **c** Mice were injected with 1 × 10^6^ MSCs (containing 5 × 10^4^ DMSCs), or 5 × 10^4^ DMSCs through tail vein. Plasma lipid was isolated at 0.5 or 4 h, and the lipid levels were detected by Nano high-resolution liquid mass analyzer. PCA plot on all samples using the normalized lipid levels of total lipids. **d**, **e** Fold change of total PS (**d**), PS (18:0/16:1)-H, PS (18:0/18:1)-H, PS (18:0/22:6)-H, PS (18:0/22:5)-H, PS (37:2)-H, and PS (39:1)-H (**e**) abundance after inj**e**ction of MSCs and DMSCs at 0.5 and 4 h. Data are represented relative to the Control group. *p < 0.05, **p < 0.01, ***p < 0.001, compared with the Control group. **c**–**e** Every dot represents one individual animal. Data are represented as mean ± SEM. *n* = 4 in each group in **b**–**e**. ANOVA with Dunnett’s multiple comparison test was performed. C-Cas 3, cleaved Caspase 3

Many studies have noted that transplanted MSCs die within a few hours, so we transferred green fluorescent protein (GFP)-MSCs into mice, to observe whether there were apoptotic MSCs. GFP-MSCs in the lung and liver tissues stayed viable within 0.5 h. However, MSCs were stained as cleaved caspase 3 positive after 2–4 h as observed by fluorescence microscopy (Fig. 4a). In addition, the numbers of living MSCs in the lung and liver were remarkably declined 12 and 24 h after GFP-MSCs injection (Fig. 4b), indicating that most transplanted GFP-MSCs died within 12 h.

As similar protective effects of MSCs and DMSCs were also observed in four tissue injury models, we next investigated what factors contributed to the protective effects of the DMSCs. Phospholipid rearrangement occurs during cell apoptosis and we determined the levels of lipids after injection of 1 × 10^6^ MSCs and 5 × 10^4^ DMSCs at 0.5 or 4 h in vivo. We found that lipid abundance varied with the injection time and cell types as analyzed by principal component analysis (PCA) (Fig. 4c). Interestingly, we found that one of the representative markers of apoptosis, PS, intensively increased after MSCs infusion. Specifically, higher levels of lipids, including PS (18:0/16:1), PS (18:0/18:1), and PS (18:0/22:6), were observed in both the DMSC-0.5 h and MSC-0.5 h groups, and a significant uplift was observed in the MSC-4 h group compared with the Control group. Furthermore, the levels of PS (18:0/22:5), PS (37:2), and PS (39:1) raised significantly 4 h after MSC injection (Fig. 4d, e). When injected the same cell number of MSCs, the total PS level did not increase at 0.5 h but significantly increased at 4 h, which suggested that PS released into the blood by apoptotic MSCs. Besides, the level of total PS in the DMSC-0.5 h group was less than that of the MSC-4 h group, indicating that the amount of released lipids correlated with the number of injected cells.

### PS protects against ConA-induced ALI and LPS-induced lung injury

**Fig. 5:**
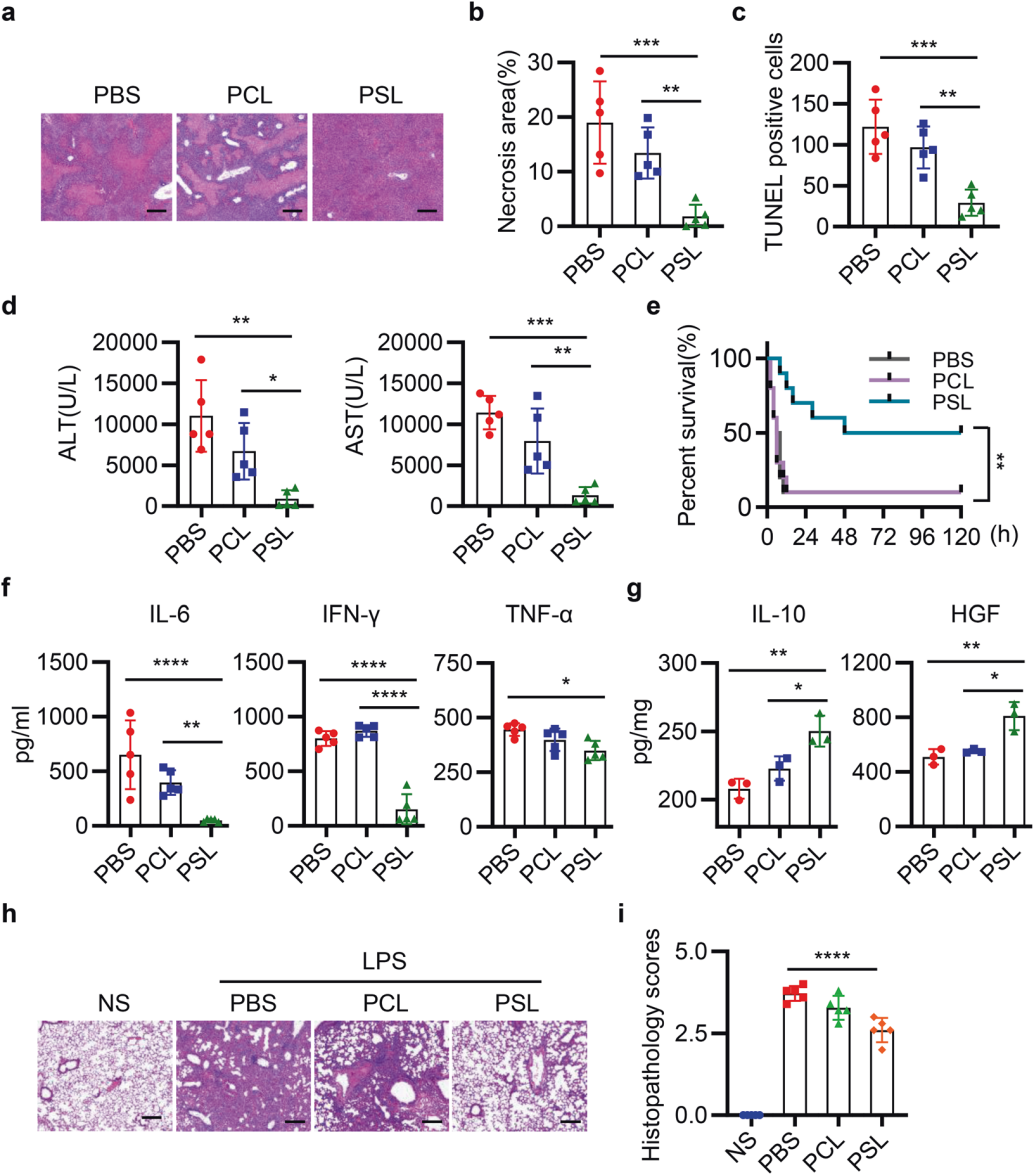
PSLs ameliorate ConA-induced ALI and LPS-induced lung injury. **a** Mice were intravenously injected with 12 mg/kg ConA, followed by treatment with PBS, PCLs, or PSLs. Representative images of liver histopathology with H&E staining in each group. Scale bar represents 200 μm. **b**–**d** Quantitative analysis of necrosis area (**b**), TUNEL-positive cells (**c**), and serum ALT and AST levels (**d**) in mice with PBS, PCLs, or PSLs treatment. *n* = 5. **e** Survival of 25 mg/kg ConA-injected mice treated with PBS, PCLs, and PSLs. *n* = 10. **f**, **g** The levels of IL-6, IFN-γ, and TNF-α in serum (**f**) and IL-10, HGF in hepatic tissues (**g**) were determined by ELISA. *n* = 3~5 in each group. **h** Representative images of lung histopathology 72 h after LPS administration. Scale bar represents 200 μm. **i** Histopathology score of lung sections in each group. *n* = 5 in each group. Data are represented as mean ± SEM. *p < 0.05, **p < 0.01, ***p < 0.001

In the next set of experiments, we investigated whether the released PS played a role in attenuating liver and lung damages. We prepared PS liposomes (PSLs) to mimic the membrane-located PS and phosphatidylcholine liposomes (PCLs) as control. Interestingly, we found that intravenous injection of PSLs exhibited hepatoprotective effects with reduced necrotic area, apoptotic hepatocytes, improved liver function, and animal survival (Fig. 5a–e). Moreover, consistent with the function of MSCs and DMSCs, PSLs also increased the levels of HGF and IL-10, and decreased the levels of proinflammatory cytokines, including IL-6, IFN-γ, and TNF-α (Fig. 5f, g). Besides, PSLs also reduced LPS-induced lung injury (Fig. 5h, i). Taken together, these findings indicated that PS exerted protective role in ALI and lung injury similar to that of MSCs and DMSCs.

### MerTK is indispensable for PS-mediated protective effects

**Fig. 6:**
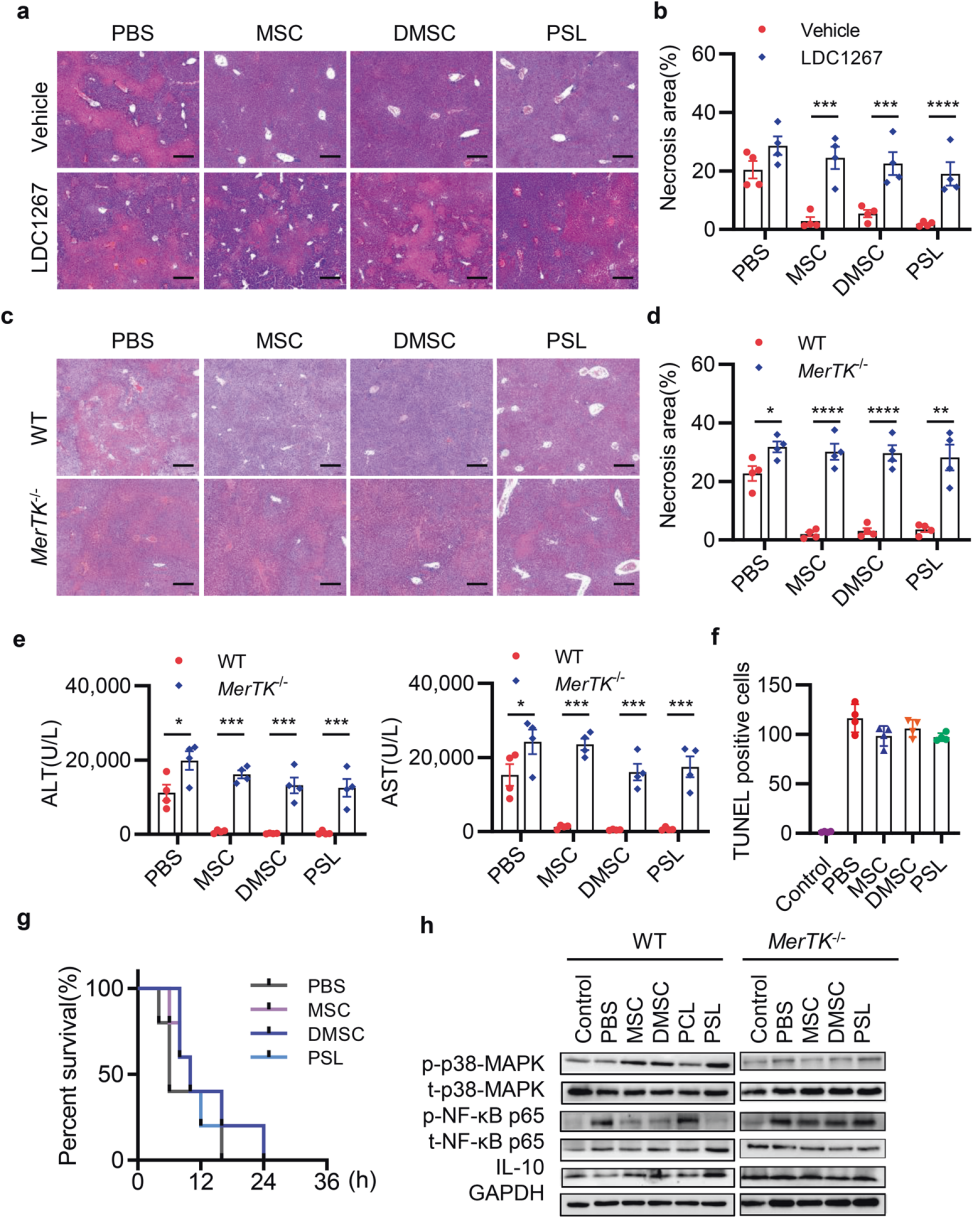
MSCs, DMSCs, and PSLs rescued ConA-induced liver injury via MerTK signaling. **a** Representative images of liver histopathology with H&E staining in ConA-injected mice treated with Vehicle and TAM receptor inhibitor LDC1267. Scale bar represents 200 μm. **b** Quantitative analysis of necrotic area in the liver of PBS-, MSCs-, DMSCs-, or PSLs-treated mice. **c** Representative H&E staining analysis of liver sections for necrosis area, inflammatory cell infiltration in WT or *MerTK*^−/−^ mice after PBS, MSCs, DMSCs, or PSLs treatment. Scale bar represents 200 μm. **d**–**f** Quantitative analysis of necrosis area (**d**), serum ALT and AST levels (**e**), and TUNEL-positive cells (**f**) in WT or *MerTK*^−/−^ mice with PBS, MSCs, DMSCs, or PSLs treatment. *n* = 4 in each group in **a**–**f**. **g** Survival of ConA-injected *MerTK*^−/−^ mice treated with PBS, MSCs, DMSCs, or PSLs. *n* = 4 in each group. **h** Protein levels of p-p38-MAPK, t-p38-MAPK, p-NF-κB p65, t-NF-κB p65, and IL-10 in each group were determined using western blotting. Data are represented as mean ± SEM. *p < 0.05, **p < 0.01, ***p < 0.001

Next, we investigated what receptors are responsible for the therapeutic effect of PSLs. Previous studies have reported that TAM (Tyro3, Axl and Mer tyrosine kinase (MerTK)) receptors are the main receptors for PS recognition expressed on monocytes/macrophages. To clarify whether MSCs, DMSCs, and PSLs protect against tissue injury through TAM receptors, we used LDC1267 to selectively inhibit MerTK, Tyro3, and Axl. LDC1267 (30 mg/kg) was injected intraperitoneally (i.p.) 30 min before ConA injection. As shown in Fig. 6a, b, administration of MSCs, DMSCs, and PSLs showed reduced necrotic areas and lower levels of AST and ALT in mice compared to the PBS group after vehicle treatment. However, pretreatment of mice with LDC1267 efficiently blocked the protective effects of MSCs, DMSCs, and PSLs, indicating that TAM receptors were crucial in mediating the therapeutic effects of MSCs, DMSCs, and PSLs. Further, we detected the mRNA expression of the three receptors. Notably, the administration of MSCs, DMSCs, and PSLs all enhanced the mRNA level of MerTK, whereas with no significant changes in the levels of Tyro3 and Axl (Supplementary Fig. S1).

To further explore whether MerTK plays an indispensable role in the immune-modulating behavior of MSCs/DMSCs/PSLs for ConA-induced liver injury, we used *MerTK*-knockout mice (*MerTK*^−/−^ group) to evaluate the therapeutic effects and B6/129 mice with the same genotype background were used as control (wild-type (WT) group). As shown in Fig. 6c–e, *MerTK*-deficient mice resulted in a more severe liver injury with visible bleeding spots, increased necrotic areas, and elevated AST and ALT levels compared with WT mice. MSCs, DMSCs, or PSLs had little effect in attenuating the liver injury in *MerTK*^−/−^ mice and failed to promote the survival in *MerTK*^−/−^ mice, when compared with that of the PBS group (Fig. 6f, g).

We next determined the molecules downstream of MerTK by western blotting in WT and *MerTK*^−/−^ mice. Levels of p-p38 mitogen-activated protein kinase (MAPK) and IL-10 were significantly increased, whereas the level of p-NF-κB p65 decreased in MSC, DMSC, and PSL groups compared with PBS and PCL groups in WT mice, but there were no significant differences in p-p38-MAPK, IL-10, or p-NF-κB p65 levels among the groups in *MerTK*^−/−^ mice (Fig. 6h). In summary, the above results suggested that MSCs, DMSCs, and PSLs ameliorate liver injury through MerTK signaling.

### MSCs reshape liver immune-microenvironment through PS or DMSCs

**Fig. 7:**
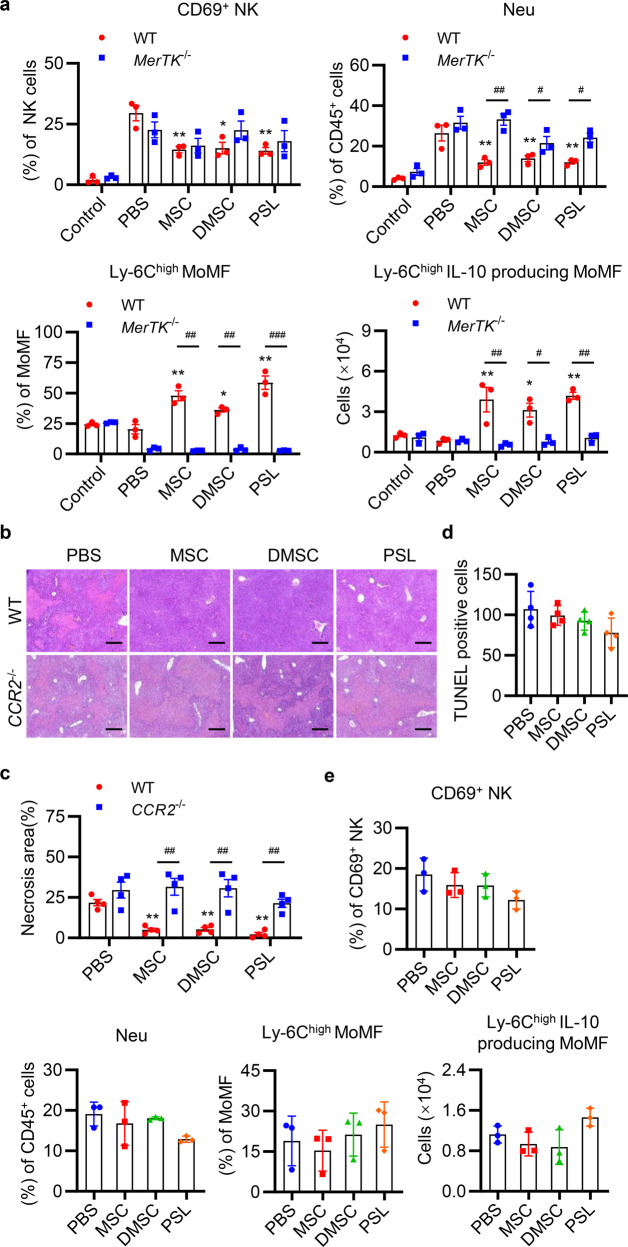
MSCs, DMSCs, and PSLs recruit Ly-6C^high^ MoMF and reduce inflammatory cell infiltration partly through CCR2. **a** Flow cytometric analyses of active NK cells (CD45^+^CD3^−^NK1.1^+^CD69^+^), neutrophils (CD45^+^CD11b^+^Ly-6G^+^), Ly-6C^high^ MoMF (CD45^+^CD11b^+^Ly-6G^−^F4/80^low^Ly-6C^high^), and total number of Ly-6C^high^ IL-10 producing MoMF in the livers of WT and *MerTK*^−/−^ mice 12 h after ConA injection followed by PBS, MSCs, DMSCs, and PSLs treatment. *n* = 3 in each group. **b** Representative H&E staining analysis of liver sections in WT or *CCR2*^−/−^ mice after PBS, MSCs, DMSCs, or PSLs treatment. Scale bar represents 200 μm. **c**, **d** Quantitative analysis of necrotic area (**c**) and TUNEL-positive cells (**d**) in liver sections of WT or *CCR2*^−/−^ mice with PBS, MSCs, DMSCs, or PSLs treatment. *n* = 4 in each group. **e** Quantitative analysis of the proportion of active NK cells, neutrophils, and Ly-6C^high^ MoMF, and the total number of Ly-6C^high^ IL-10 producing MoMF in the livers of *CCR2*^−/−^ mice by flow cytometry. *n* = 3 in each group. Data are represented as mean ± SEM. *p < 0.05, **p < 0.01, ***p < 0.001, compared with PBS group in WT mice. ^#^*p* < 0.05, ^##^*p* < 0.01, compared with each other

To further understand which population of immune cells were effective in the process of liver protection, we evaluated the activation of CD4^+^ T cells, CD8^+^ T cells, and natural killer (NK) cells. The infiltration of neutrophils, monocyte-derived macrophages (MoMF), and kupffer cells in liver tissues were also characterized. The gating strategy for immune cells in the liver is shown in Supplementary Fig. S2a, b referring to the previous reports.^27–29^ As shown in Fig. 7a, in contrast to mice treated with PBS, mice treated with MSCs, DMSCs, or PSLs showed a significant reduction in the percentages of activated NK cells and infiltrated neutrophils. There were no significant differences in the percentages of activated CD4^+^ T cells, CD8^+^ T cells (data not shown), and the percentage of Ly-6C^high^ MoMF, and number of Ly-6C^high^ IL-10-producing MoMF were significantly increased in MSC, DMSC, and PSL groups compared to that of the PBS group. Moreover, these changes were reversed in *MerTK*^−/−^ mice.

### DMSCs and PSLs recruit Ly-6C^high^ IL-10-producing MoMF partly through CCR2

From the above results, we knew that MSCs, DMSCs, and PSLs accelerated the recruitment of Ly-6C^high^ MoMF. To clarify the function of this cell population in MSCs, DMSCs, and PSLs regulated liver recovery, we detected the hepatoprotective effects of MSCs, DMSCs, and PSLs in *CCR2*^−/−^ mice, as previous studies demonstrated that monocytes recruitment mainly depended on C–C motif chemokine receptor 2 (CCR2) in injured liver tissue.^27,30^ The results showed that MSCs, DMSCs, and PSLs attenuated liver injury compared with the PBS group in WT mice, but these effects were eliminated in *CCR2*^−/−^ mice (Fig. 7b–d). Moreover, the percentages of activated NK cells and infiltrated neutrophils or Ly-6C^high^ MoMF showed no significant differences among all the groups (Fig. 7e). These results suggested that CCR2 played an important role in the MSCs-, DMSCs-, and PSLs-mediated reduced neutrophil infiltration and promoted Ly-6C^high^ MoMF recruitment.

### DMSCs and PSLs promote macrophages polarization into M2 phenotypes

In addition to the function in immune cell recruitment, we also paid attention to how DMSCs regulate cell phenotype by releasing PS. As the apoptotic cells exerted anti-inflammatory property, we investigated whether PS could induce the macrophages into an immunosuppressive M2 phenotype by evaluating the expression levels of F4/80, inducible nitric oxide synthase (iNOS), and CD206. Notably, the treatment of MSCs, DMSCs, and PSLs decreased the percentages of F4/80^+^iNOS^+^ macrophages (M1 macrophages) and increased the percentages of F4/80^+^CD206^+^ macrophages (M2 macrophages) (Supplementary Fig. S3a, b). The detected mRNA levels of iNOS and CD206 in the liver tissues were also consistent with the immunofluorescence results (Supplementary Fig. S3c). Moreover, MSCs, DMSCs, and PSLs induced higher level of IL-10 in macrophages (Supplementary Fig. S3d). In conclusion, MSCs, DMSCs, and PSLs could promote monocytes/macrophages polarization into M2 phenotypes with enhanced IL-10 secretion.

## Discussion

In the current study, several observations have been made concerned with MSC therapy, spontaneous cell death, and PS exposure. MSC-based cell therapy is reported to be effective in several acute inflammatory models.^1–6^ Here we investigated the immune-modulating mechanisms and elucidated that the apoptotic MSCs in a therapeutic preparation played a critical role in attenuating the tissue injury. The conclusion is supported by the following evidence. We found that upon the injection of living MSCs, most transplanted MSCs underwent apoptosis within 12 h. In addition to this, treatment of MSCs or pure apoptotic MSCs (DMSCs) had similar protective effects in four mouse injury models. In the next set of experiments, we observed a significant increase in the level of PS after MSCs or DMSCs treatment. Surprisingly, the injection of PS alone also exerted a protective effect in ALI and LPS-induced lung injury, which is similar to that of MSCs and DMSCs. Furthermore, the administration of MSCs and DMSCs enhanced the mRNA level of *MerTK*, which is a PS receptor, and the hepatoprotective effects of MSCs, DMSCs, and PSLs were diminished in treating the *MerTK*^−/−^ ALI mouse model. Moreover, the results of flow cytometry suggested that MSCs or DMSCs could alter the cell populations in microenvironment and recruit more Ly-6C^high^ IL-10-producing MoMF through CCR2 by releasing PS, which is a key population of the cells to exert an anti-inflammatory property. In addition, it is found that PS released by DMSCs could regulate cell phenotype by inducing the macrophage into an immunosuppressive M2 phenotype. To summarize, our results indicated that MSCs therapy exerted a protective effect through the release of PS from spontaneous DMSCs, which subsequently activated MerTK and reshaped the immune-microenvironment by the recruitment and induction of anti-inflammatory cell phenotypes.

MSCs exert an effective therapeutic effect on several acute injury models,^31,32^ but the detailed mechanisms of its immunomodulation are not fully understood. Especially, when it comes to the role of apoptotic MSCs in immune regulation after MSCs transplantation, many questions remain. Previous reports support the implanted cells easily go through apoptosis.^33,34^ For instance, Bishop et al.^33^ found that most intravenously (i.v.) injected tumor cells died within 7 h. Eggenhofer et al.^34^ noted that MSCs were short-lived and could not migrate beyond the lungs after intravenous infusion, which suggests that MSCs exert long-term immunomodulatory and regenerative effects that might be mediated via other components. In addition, from preclinical and clinical studies, we concluded that viable cells account for 50~95% of the cells in MSC infusions,^1,6^^,^^35–37^ which indicates that nonviable cells may be effective components. In the current study, we discovered that equal number of DMSCs (5 × 10^4^ DMSCs) in our DMSC-containing MSC preparation were enough to exert protective effects in ConA- and CCl_4_-induced ALI, LPS-induced lung injury, and SCI. This indicates that DMSCs could exhibit equal protective effects as MSCs and exhibit an immunomodulatory capacity. However, injection of H_2_O_2_-induced apoptotic MSCs was invalid (data not shown). This phenomenon was consistent with the previous study that chemically induced apoptotic MSCs could not improve survival or decrease severity of endotoxin-induced lung injury.^38^ This could be explained by the high level of oxidative stress in such apoptotic MSCs.^39^ In summary, these results indicate that MSCs undergoing spontaneous apoptosis but not induced death could exert an immunomodulatory ability.

As the most abundant negatively charged glycerophospholipid in eukaryotic plasma membranes,^40^ externalized PS functions as a dominant immunosuppressive factor.^22^ Here we revealed that intravenous fusion of MSCs and DMSCs increased plasma PS level. In addition, the injection of PS alone (PSLs) displayed an efficient anti-inflammatory activity similar to that of MSCs and DMSCs. As the major receptor of PS on monocytes/macrophages, TAM receptors are involved in dampening innate immune responses and promoting the clearance of apoptotic cells.^41^ We found that the TAM receptor inhibitor LDC1267 abolished the hepatoprotective effects of MSCs, DMSCs, and PSLs. Interestingly, an obvious change in mRNA levels was observed for MerTK but not for Axl or Tyro3. MerTK activation is involved in the suppression of inflammation, synthesis of inflammatory mediators, and promotion of apoptotic cell clearance following acute tissue damage during tissue repair.^41–44^ Triantafyllou et al.^41^ found that MerTK-expressing hepatic macrophages promote the resolution of inflammation in acetaminophen (APAP)-induced ALI, and that APAP-treated *MerTK*^−/−^ mice exhibit persistent liver injury and inflammation. Consistent with the function of MerTK in APAP-induced ALI, we found that *MerTK* deficiency led to ConA-induced ALI deterioration. Moreover, MerTK was found to play an indispensable role in the hepatoprotective effects of MSCs, DMSCs, and PSLs in ALI. Therefore, our study addressed the role of DMSCs in immunomodulation and elucidated a new mechanism of MSCs attenuating tissue injury through the release of PS from DMSCs, which involved MerTK activation. The results also suggested that MerTK is an important regulatory molecule for tissue repair in an ALI model.

Previous studies have shown that MSCs exert immunomodulatory functions and could regulate monocytes/macrophages, dendritic cells, T cells, B cells, and NK cells.^45–47^ Among them, anti-inflammatory monocytes/macrophages play a prominent role in the complex interactions mediated by MSCs.^48^ Here we found that Ly-6C^high^ MoMF recruitment was correlated with recovery from liver injury. Importantly, the Ly-6C^high^ MoMF population with high expression of CX3CR1 produced the cytokine IL-10, which indicates that these Ly-6C^high^ CX3CR1^+^ MoMF may be distinguished from conventional proinflammatory Ly-6C^high^ MoMF (identified as CX3CR1^low^CCR2^high^). Such findings are also in accordance with the previous reports that monocytes with high expression of CX3CR1 are functional in tissue repair.^49^ In addition to the regulation of MoMF population, MSCs induced monocytes/macrophages polarization into M2 macrophages and promoted IL-10 production. The key mediator for macrophage M2 polarization is PS from DMSCs, which also throw some light on how M2 macrophages participate in the tissue repair process in MSC therapy.

In conclusion, our data elucidate that the apoptotic MSCs (DMSCs) in a therapeutic preparation play the partial role in attenuating the tissue injury by exposing PS. PS from the DMSCs activates MerTK and increases the IL-10-producing Ly-6C^high^ MoMF recruitment through CCR2. In addition to the decreased neutrophil infiltration, the inhibition of NK cell activation, PS from DMSCs, also induces macrophage polarization towards M2 phenotype. These results provide a new insight how MSC-based therapies function and exert an immunomodulating property in the acute tissue injury model.

## Materials and methods

### Animals

Female C57BL/6 mice were purchased from Vital River (Beijing, China) and bred in a specific pathogen-free environment with consistent room temperature and humidity. B6.129-MerTK^tm1Gr1^/J (*MerTK*^−/−^), B6.129S4-Ccr2^tm1Ifc^/J (*CCR2*^−/−^), and B6J.129(Cg)-Gt (ROSA)26Sor^tm1.1 (CAG-cas9*, -EGFP) Fezh^/J (GFP) mice were obtained from The Jackson Laboratory (USA). B6.129SF2/J mice with an identical background as the *MerTK*^−/−^ mice were obtained by breeding C57BL/6J females and 129S1/SvImJ males (Vital River, Beijing, China). All animal experiments were approved by the Institutional Animal Care and Use Committee.

### Isolation of bone marrow-derived MSCs

MSCs were isolated from C57BL/6 female mice, aged 2–3 weeks, as described previously.^23^ In brief, the femurs and tibias of donor mice were isolated, and the bone marrow was flushed with Hank’s buffered saline solution and centrifuged at 700 × *g* for 5 min. The bones were crushed into chips of ~1–3 mm^3^ with scissors and incubated for 1 h at 37 °C in 5 ml Dulbecco’s modified Eagle’s medium (DMEM) containing 10% fetal bovine serum (FBS) in the presence of 1 mg/ml collagenase II. Then, the collected flushed cells and enzyme-treated bone chips were cultured in DMEM (Gibco, USA) supplemented with 10% FBS and 100 U/ml penicillin/streptomycin at 37 °C in 5% CO_2_. Two days later, the nonadherent cells were removed and the adherent cells were washed once with PBS and then cultured with supplemented medium. MSCs in passages three to six were used in this study.

### Characterization of MSCs

MSCs were collected and resuspended in 100 μl PBS. Then, the MSCs were stained with PerCP/Cy5.5-conjugated anti-mouse CD45 antibody, Brilliant Violet 711-conjugated anti-mouse CD11b antibody, Phycoerythrin (PE)-conjugated anti-mouse CD31 antibody, fluorescein isothiocyanate (FITC)-conjugated anti-CD86 antibody, FITC-conjugated anti-mouse CD29 antibody, PE-conjugated anti-mouse CD44 antibody, and Allophycocyanin (APC)-conjugated anti-mouse Sca-1 antibody with the corresponding isotype as control (BioLegend, USA) for 30 min at 4 °C in the dark. MSC surface markers were analyzed by flow cytometry.

### Identification of MSCs and DMSCs

The viability of MSCs was determined by Trypan blue staining. In general, 90 μl cell suspension and 10 μl 0.4% trypan blue solution were mixed, and the proportion of living cells was calculated. DMSCs were defined as the cells that spontaneously died during MSC culture and were collected by centrifugation at 800 × *g* for 5 min. Accurate counts of live MSCs and DMSCs were recorded by flow cytometry with cell staining using LIVE/DEAD™ Fixable Near-IR Dead Cell Stain Kit (Thermo Fisher, USA).

### Induction of ALI and cell transplantation

Eight- to 10-week-old C57BL/6 mice were used for ALI induction by ConA or CCl_4_. For ConA-induced acute injury, ConA dissolved in sterile saline (12 mg/kg body weight (BW)) was injected into WT, *MerTK*^−/−^, and *CCR2*^−/−^ mice via the tail vein (i.v.) according to previous studies.^50^ For CCl_4_-induced acute injury, a single dose of CCl_4_ (5% vol/vol in corn oil, 0.5 ml/kg BW) was i.p. injected into mice. Then, MSCs or DMSCs were administered i.v. within 30 min after ConA or CCl_4_ injection. All mice were killed 12 h after ConA injection or 24 h after CCl_4_ injection. Blood samples were collected and the serum was extracted. Liver tissues were collected for histopathological analysis and biochemical studies.

For the MSC- or DMSC-treated experimental groups: after ConA or CCl_4_ injection, MSCs were suspended in PBS at 1 × 10^6^ cells in a final volume of 100 μl per mouse. Each preparation of MSCs approximately contains 5% DMSCs (~5 × 10^4^ DMSCs), which was confirmed by flow cytometry. For the DMSCs-treated experimental groups, the collected DMSCs (5 × 10^4^, 1 × 10^5^, 2.5 × 10^5^, 5 × 10^5^, and 1 × 10^6^ cells per mouse) were injected via tail vein in a final volume of 100 μl.

### Lung injury model

The intratracheal instillation was carried out as described previously.^51^ Briefly, mice were anesthetized by 5% isoflurane and followed by instillation of LPS (5 mg/kg) or normal saline in 50 μl volume. Then MSCs (1 × 10^6^ cells containing ~5 × 10^4^ DMSCs per mouse) or DMSCs (5 × 10^4^ cells per mouse) were injected into mice via tail vein in a final volume of 100 μl. Mice were killed 72 h after LPS instillation. The lung tissues were collected for hematoxylin and eosin (H&E) staining to assess lung pathology.

### Spinal cord injury

The spinal cord contusion injury was performed as described previously.^52^ Briefly, mice were anesthetized with an i.p. injection of 1% pentobarbital sodium. The mice skin was incised along the midline of the back and the muscles at the thoracic level (T9–T11) were dissected out. A laminectomy at the tenth thoracic level was performed and the spinal cord was hit using an Infinite Horizons Impactor (Precision Systems Instrumentation, Lexington, CA). After injury, the skin was closed with wound clips, the mice were injected (subcutaneously) with penicillin, and placed in a temperature-controlled pad to recover until thermoregulation was re-established. Then MSCs (1 × 10^6^ cells containing ~5 × 10^4^ DMSCs per mouse) or DMSCs (5 × 10^4^ cells per mouse) were injected into mice via tail vein in a final volume of 100 μl. The mice were provided with soft food and water. Bladder expression was performed twice daily until the recovery of bladder control.

### Behavioral assessment

The hindlimb locomotor recovery and motor function was assessed according to BMS open-field score.^53^ A team of tow experienced examiners evaluated each mouse for 4 min and gave a score for each hindlimb. BMS test was performed at 1, 3, and 7 days postoperative and weekly thereafter.

### Survival analysis

For survival analysis, 25 mg/kg ConA or 5 ml/kg CCl_4_ was injected into mice. PBS, MSCs, DMSCs, PSLs, or PCLs were administered i.v. within 30 min after ConA or CCl_4_ injection.

### Histopathology

Collected liver and lung tissues were immediately fixed with 4% paraformaldehyde and embedded in paraffin. Four-micrometer-thick sections of tissues were stained with H&E to evaluate histopathologic damage. The whole slides were scanned digitally with the Pannoramic MIDI scanner (3DHISTECH, Hungary). The necrotic area of liver tissues was examined by independent pathologists and the lung injury scores were evaluated by three investigators who were blinded to the experiments following a previously published scoring system^54^.

### Serum aminotransferase analysis

The levels of AST and ALT in the serum were detected using Cobas 4000 analyzer series (Roche, Basel, Switzerland). Enzyme activities are expressed in international units (U/L).

### Flow cytometry

Staining of liver-infiltrating mononuclear cells was performed as previously described.^50,55^ Briefly, mice were killed and the liver was perfused with 30 ml cold PBS, to eliminate circulating leukocytes. Then, the liver was digested with collagenase type IV (Gibco, USA) at 37 °C for 30 min. Hepatic mononuclear cells were stained for cell-surface markers at 4 °C for 30 min before detection. Specifically, PerCP/Cy5.5-conjugated anti-mouse CD45, FITC-conjugated anti-mouse CD11b, Brilliant Violet 510-conjugated anti-mouse Ly-6G, APC-conjugated anti-mouse F4/80, PE-conjugated anti-mouse Ly-6C, PE-conjugated anti-mouse CD45, PerCP/Cy5.5-conjugated anti-mouse CD3, APC-conjugated anti-mouse CD4, Brilliant Violet 510-conjugated anti-mouse CD8, Brilliant Violet 650-conjugated anti-mouse NK1.1, FITC-conjugated anti-mouse CD69, APC-conjugated anti-mouse Ly-6C, PE-conjugated anti-mouse CX3CR1, and Brilliant Violet 650-conjugated anti-mouse CCR2 antibodies from BioLegend (USA) were used. For intracellular staining for IL-10, cells were incubated with GolgiStop (BD Bioscience, USA) for 4 h and stained for surface markers. Then, the cells were fixed and permeabilized with BD Cytofix/Cytoperm^TM^ Fixation/Permeabilization Kit (BD Bioscience, USA) and PE-Cy7-conjugated anti-mouse IL-10 antibody (BioLegend, USA) was added for staining at room temperature for 1 h. Samples were washed and evaluated on ACEA NovoCyte^TM^ system. Dead cells were excluded using 4′,6-diamidino-2-phenylindole staining (D1306, Life Technologies)^56^ and Precision Count Beads™ (BioLegend, USA) were used for quantification of absolute cell numbers. Data were analyzed with NovoExpress^TM^ software.

### Cytokine quantification by enzyme-linked immunosorbent assay

The levels of IL-6, TNF-α, and IFN-γ in serum, and IL-10 and HGF in liver tissues were determined using enzyme-linked immunosorbent assay (ELISA) kits according to the manufacturer’s instructions. The HGF ELISA kit was purchased from R&D Systems and the others were purchased from Thermo Fisher.

### TUNEL assay

TUNEL (terminal deoxynucleotidyl transferase dUTP nick end labeling) assay was carried out by using DeadEnd^TM^ Fluorometric TUNEL System (Promega, USA). The results were observed by fluorescence microscope (Leica, Germany). The number of positive cells were calculated from observation of five random fields.

### RNA isolation and real-time quantitative PCR

Total RNA was isolated from liver tissues using Animal Total RNA Isolation Kit (Foregene Company, Chengdu, China) according to the manufacturer’s instructions. Total RNA (500 ng) was transcribed using the PrimeScript^TM^ RT Master Mix Kit (TAKAR, Dalian, China). Real-time reverse-transcriptase PCR was carried out in triplicate using a Bio-Rad CFX Connect PCR system (Bio-Rad, USA). The sequences of the primers used are shown in Supplementary Table S2. β-Actin was used as internal reference gene and 2^−ΔΔCt^ method was applied to analyze data.

### Immunofluorescence staining

DMSCs were attached to slides by centrifugation at 300 r.p.m. for 5 min using Shandon Cytospin 4 (Thermo Fisher, USA). Frozen sections (5 μm) of the liver tissue were used for immunofluorescence staining. Sections were fixed with cold methanol for 10 min and treated with Triton-X 100 for 10 min. Then, they were blocked with donkey serum or goat serum for 30 min at room temperature. Subsequently, sections were incubated with goat anti-CD206 antibody (1 : 200, R&D), rabbit anti-iNOS antibody (1 : 400, Abcam), rat anti-F4/80 antibody (1 : 200, Abcam), rabbit anti-cleaved caspase 3 (1 : 400, Cell Signaling Technology), or rabbit anti-Cathepsin B (1 : 200, Abcam) overnight at 4 °C. Then, sections were incubated with F488-labeled goat anti-rabbit secondary antibody (1 : 400, Thermo Fisher), 555-labeled goat anti-rat secondary antibody (1 : 400, Thermo Fisher), or F488-labeled donkey-anti-goat secondary antibody (1 : 400, Thermo Fisher) for 1 h at room temperature. The results were observed with a fluorescence microscope (Leica, Germany).

### Western blot analysis

Total protein was isolated from liver tissues, and 30 μg protein was separated by SDS-polyacrylamide gel electrophoresis and transferred to a polyvinylidene difluoride membrane. After blocking with 5% non-fat milk, membranes were incubated with rabbit anti-IL-10 (1 : 1000), rabbit anti-p38-MAPK (1 : 1000), rabbit anti-p-p38-MAPK (1 : 1000), rabbit anti-NF-κB p65 (1 : 1000), rabbit anti-p-NF-κB p65 (1 : 1000), and mouse anti-GAPDH (1 : 2000) antibodies at 4 °C overnight. Then, membranes were incubated with horseradish peroxidase-labeled goat anti-rabbit (1 : 5000) and goat anti-mouse (1 : 10,000) secondary antibodies (Santa Cruz, USA). Results were visualized using ChemiDoc Touch (Bio-Rad, USA).

### Isolation and detection of lipids from plasma

Mouse plasma (100 μl) was fully mixed by vortexing with 400 μl CH_2_Cl_2_ : MeOH (2 : 1) solution. Then, the mixture was centrifuged at 3000 r.p.m. for 15 min at 4 °C. The lower organic phase was removed and mixed with a quarter volume of ddH_2_O. Then, the mixture was centrifuged at 10,000 r.p.m. for 20 min at 4 °C. The lower phase was extracted and dried under N_2_ gas. 1-Palmitoyl-d31-2-oleoyl-sn-glycero-3-phosphoethanolamine (16:0-d31-18:1 PE; Avanti, USA) was used as an internal reference. The lipid content was detected with a Nano high-resolution liquid mass analyzer. To identify the distances between samples based on levels of released lipids, raw data were normalized by counts function implemented in DESeq2 package. Then, PCA was used to calculate the rotation matrix based on data processed by prcomp function in the Stats package. The PCA plot was visualized by ggplot2 package with modified codes.

### Preparation and characterization of liposomes

Liposomes were prepared according to previous studies.^57^ Liposomes were composed of PC (840053, Avanti, USA), PS, and cholesterol (CH) at a molar ratio of 2 : 1 : 1 (PSLs) or PC and CH at a 3 : 1 molar ratio (PCLs). Briefly, the phospholipids and CH were dissolved in ethyl alcohol. The solvent was evaporated and samples were dried under N_2_ gas for 3 h, resuspended in PBS, and sonicated for 3 min at 4 °C. Finally, the solution was passed through a membrane filter (0.45 μm, Corning Glassworks, Corning, NY) before use. Liposomal size and Zeta potential were determined by dynamic light scattering using Malvern Zetasizer (Malvern Instruments, Malvern, UK). The mean diameter and Zeta potential of PSLs and PCLs were ~231 nm, −40 mV and 208 nm, −5 mV, respectively. Liposomes (1 µM total lipids) were i.v. injected into mice immediately after ConA or LPS challenge.

### Endotoxin detection

The endotoxin content in PSLs and PCLs was detected by Tachypleus Amebocyte Lysate Kit (Xiamen Bioendo Technology Co., Xiamen, China) according to the manufacturer’s instructions. Liposomes with endotoxin level <0.25 EU/ml were used through the study.

### Statistical analysis

The data were presented as mean ± SEM and were analyzed by SPSS software 19.0. Student’s two-tailed *t*-test was used to study the possible association between two variables, one-way analysis of variance was used to analyze differences among multiple comparisons, and Student–Newman–Keuls test used for comparisons of each other. Animal survival was analyzed by log rank tests and *p*-values are shown. *p* < 0.05 was considered significant.

## Supplementary information

supplementary material figures

## Data Availability

The authors declare that there are no primary datasets and computer codes associated with this study. All data and materials are available to the researchers once published.
