# Correction: Spontaneous apoptosis of cells in therapeutic stem cell preparation exert immunomodulatory effects through release of phosphatidylserine

**DOI:** 10.1038/s41392-021-00862-3

**Published:** 2022-01-13

**Authors:** Xuemei He, Weiqi Hong, Jingyun Yang, Hong Lei, Tianqi Lu, Cai He, Zhenfei Bi, Xiangyu Pan, Yu Liu, Lunzhi Dai, Wei Wang, Canhua Huang, Hongxin Deng, Xiawei Wei

**Affiliations:** 1 Laboratory of Aging Research and Cancer Drug Target, State Key Laboratory of Biotherapy and Cancer Center, National Clinical Research Center for Geriatrics, West China Hospital, Sichuan University, Chengdu, Sichuan People’s Republic of China; 2 Experimental Medicine Center, the Affiliated Hospital of Southwest Medical University, Luzhou, Sichuan People’s Republic of China

**Keywords:** Immunological disorders, Inflammation

Correction to: *Sig Transduct Target Therapy* 10.1038/s41392-021-00688-z, published online 14 July 2021

After online publication of the article^1^, the authors noticed one inadvertent mistake in Fig. 5a that needs to be corrected. In detail, the pathological picture of PBS group in Fig. 5a is inadvertently duplicated as the image of PBS group in Fig. 7b in the main text. This duplication is a result of errors in figure assembly, and the correct Fig. 5 is provided as follows. The key findings of the article are not affected by these corrections.

**Fig. 5:**
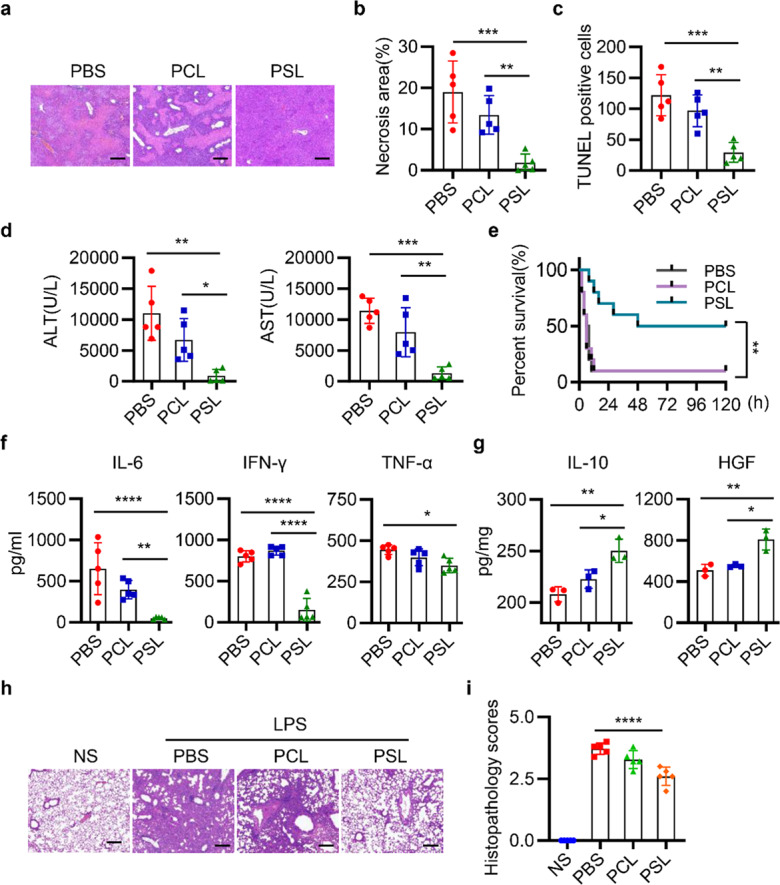
PSLs ameliorate ConA-induced ALI and LPS-induced lung injury. **a** Mice were intravenously injected with 12 mg/kg ConA, followed by treatment with PBS, PCLs, or PSLs. Representative images of liver histopathology with H&E staining in each group. Scale bar represents 200 μm. **b**–**d** Quantitative analysis of necrosis area (**b**), TUNEL-positive cells (**c**), and serum ALT and AST levels (**d**) in mice with PBS, PCLs, or PSLs treatment. *n* = 5. **e** Survival of 25 mg/kg ConA-injected mice treated with PBS, PCLs, and PSLs. *n* = 10. **f**, **g** The levels of IL-6, IFN-γ, and TNF-α in serum (**f**) and IL-10, HGF in hepatic tissues (**g**) were determined by ELISA. *n* = 3~5 in each group. **h** Representative images of lung histopathology 72 h after LPS administration. Scale bar represents 200 μm. **i** Histopathology score of lung sections in each group. *n* = 5 in each group. Data are represented as mean ± SEM. *p < 0.05, **p < 0.01, ***p < 0.001
