# Supplementary material for: Spontaneous apoptosis of cells in therapeutic stem cell preparation exert immunomodulatory effects through release of phosphatidylserine
Source: Signal Transduct Target Ther. 2021 Jul 14;6:270. doi: 10.1038/s41392-021-00688-z (PMC8280232; doi:10.1038/s41392-021-00688-z)
Supplement: Supplementary file 1 — supplementary material figures [file 41392_2021_688_MOESM1_ESM.docx]

Supplementary Materials for

Spontaneous apoptosis of cells in therapeutic stem cell preparation exert immunomodulatory effects through release of phosphatidylserine

Xuemei He^1,2#^, Weiqi Hong^1#^, Jingyun Yang^1#^, Hong Lei^1^, Tianqi Lu^1^, Cai He^1^, Zhenfei Bi^1^, Xiangyu Pan^1^, Yu Liu^1^, Lunzhi Dai^1^, Wei Wang^1^, Canhua Huang^1^, Xiawei Wei^1*^

Correspondence to: xiaweiwei@scu.edu.cn

**This PDF file includes:**

Figures. S1 to S3

Tables S1 to S2

Figure. S1.


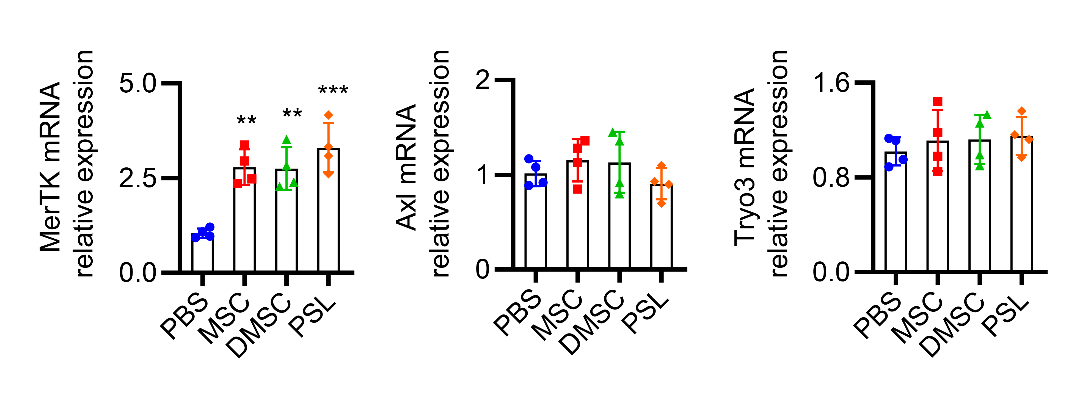


**Figure S1. MerTK mRNA level was elevated in liver after MSCs, DMSCs and PSLs treatment.** RT-qPCR was used to analyzed the mRNA levels of MerTK, Axl and Tyro3. Data are represented as mean ± SEM. **p < 0.01, *** p < 0.001, compared with PBS group.

Figure. S2.


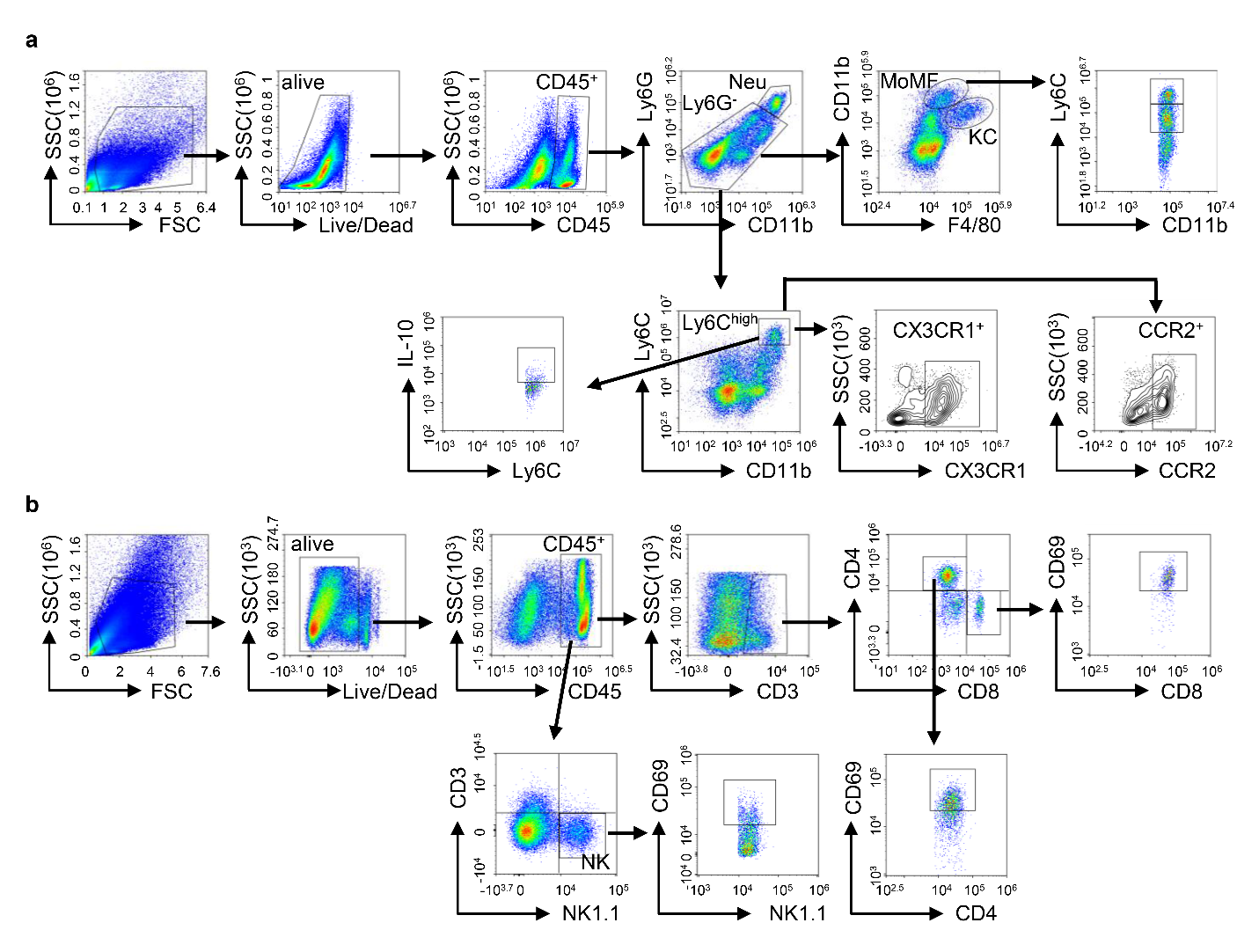


**Figure S2. Gating strategy and flow cytometry analysis of immune cells in liver tissues.**

**a** Neutrophils were identified as CD45^+^CD11b^+^Ly-6G^+^ cells. Monocyte-derived macrophages (MoMF) were identified as CD45^+^CD11b^+^Ly-6G^-^F4/80^low^, and resident kupffer cells (KCs) were identified as CD45^+^CD11b^-^Ly-6G^-^F4/80^+^. **b** NK cells in liver tissues were identified as CD45^+^CD3^-^NK1.1^+^ cells. T cells were identified as CD45^+^CD3^+^, either CD4^+^ or CD8^+^ cells. Activated cells were further differentiated as CD69^+^.

Figure. S3.


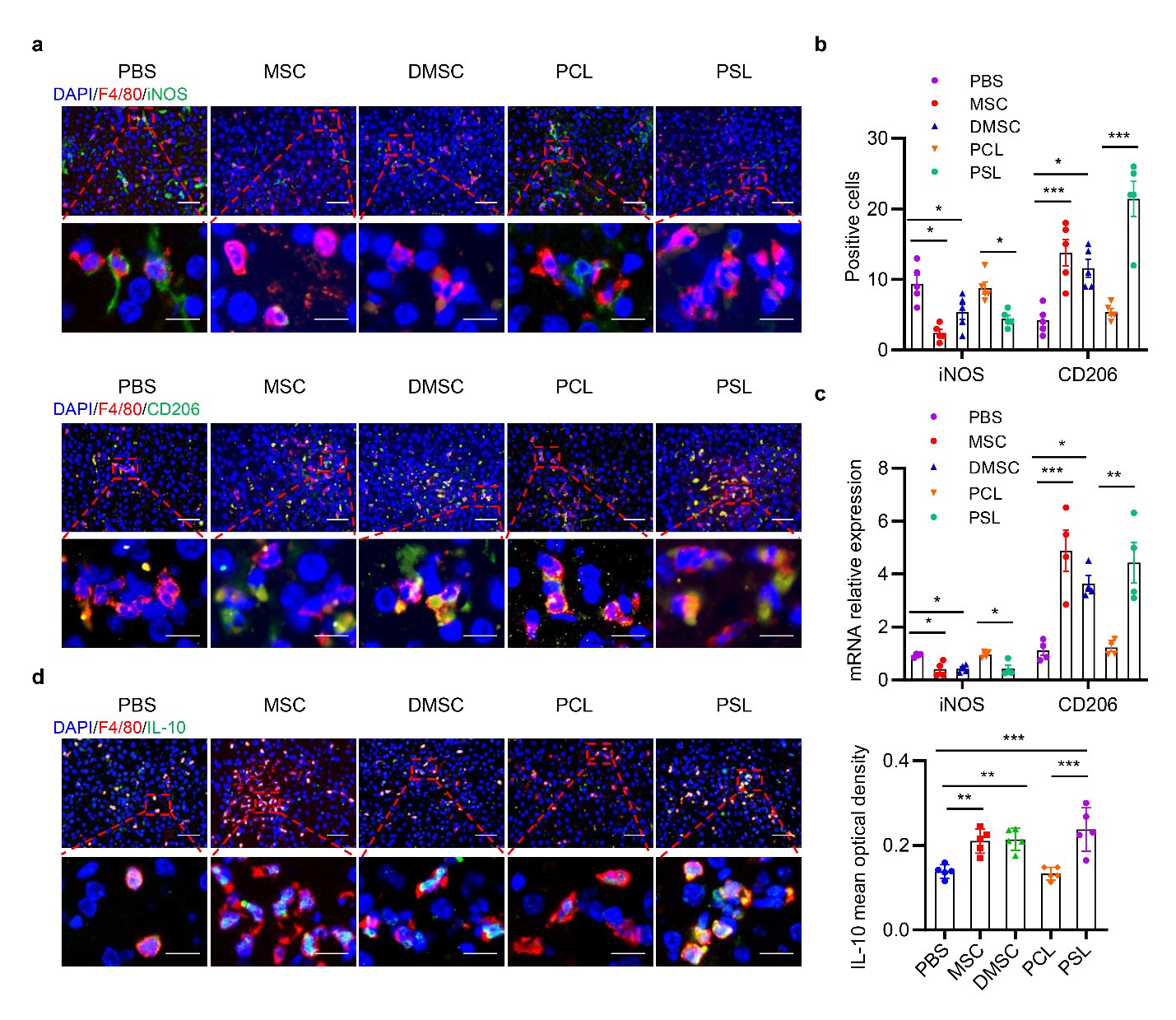


**Figure S3. MSCs, DMSCs and PSLs promote monocytes/macrophages polarized to M2 in mice. a** Immunofluorescence staining analysis of M1 macrophage (top) with markers for F4/80 (red), iNOS (green) and DAPI (blue), and M2 macrophage (bottom) with makers for F4/80 (red), CD206 (green) and DAPI (blue) in mouse liver sections. Scale bar represents 50 μm (upper row) or 10 μm (lower pannel). **b** Quantitative analysis of the M1 and M2 macrophages counts.

**c** mRNA expression of iNOS and CD206 in livers of PBS, MSCs, DMSCs or PSLs treated mice were determined by qPCR. **d** Immunofluorescence staining analysis of F4/80 (red) macrophage with expression of IL-10 (green). Right panel, quantitative analysis of the macrophage expressed IL-10 counts. Scale bar represents 50 μm (upper row) or 10 μm (lower pannel). Data are represented as mean ± SEM. *p < 0.05, **p < 0.01, ***p < 0.001, compared with PBS group.

Table S1.

**Table S1 Count of dead cells in MSC preparation in clinical studies**

| **Disease** | **ClinicalTrials.gov Identifier** | **Age (years)** | **Injection position** | **Number** | **Phase** | **Cell source** | **Cell number** | **Viability (%)** | | **References** |
| --- | --- | --- | --- | --- | --- | --- | --- | --- | --- | --- |
| ALS | NCT01363401 | 25~75 | intrathecal delivery | 64 | 1/2 | BMSC | 1×10^6^ cells/kg | 94%~95% | ^1^ | |
| Acute M/I | NCT00114452 | 21~85 | i.v. | 53 | I | BMSC | 0.5/1.6/5×10^6^/kg | ≥70% | ^2^ | |
| ARDS | NCT01775774 | ≥18 | i.v. | 9 | 1 | MSC | 1/5/10×10^6^ cells/kg | 50%-63% | ^3^ | |
| Multiple Sclerosis | NCT02239393 | 18~50 | i.v. | 31 | II | MSC | 1/2×10^6^ cells/kg | >80% | ^4^ | |
| Alcoholic Liver Cirrhosis | NCT01875081 | 20~70 | hepatic artery | 72 | II | MSC | 5×10^7^ cells | >70% | ^5^ | |
| Multiple Sclerosis | NCT02403947 | 18~50 | i.v. | 1 | I/II | MSC | 1/2×10^6^ cells/kg | >80% | ^4^ | |
| Knee Osteoarthritis | NCT02580695 | 18~70 | i.a. | 30 | I/II | UC-MSC | 2×10^7^ cells | >80% | ^6^ | |
| Osteoarthritis | NCT02351011 | 40~65 | i.a. | 12 | I/II | BMSC | 1/10/50×10^6^ cells | >70% | ^7^ | |
| GVHD | 000024291 | 20~74 | intra-bone marrow cavity | 6 | I | BMSC | 5×10^7^ cells | >70% | ^8^ | |
| Acute Respiratory Distress Syndrome | NCT02097641 | ≥18 | i.v. | 60 | II | BMSC | 1×10^7^ cells | 36%~85% | ^9^ | |
| Dilated Cardiomyopathy | NCT01739777 | 18~75 | i.v. | 30 | I/II | UC-MSC | 1×10^6^ cells/kg | >80% | ^10^ | |
| Patellar Tendinopathy | NCT03454737 | 18~48 | tendinous and patellar tendon areas | 20 | I/II | MSC | 2×10^7^ cells | >90% | ^11^ | |
| Degenerative Arthritis | NCT 02658344 | ≥18 | i.a. | 24 | II | ADMSC | 1×10^8^ cells | ≥87% | ^12^ | |
| Severe Intraventricular Hemorrhage | NCT02274428 | 0.5~0.7 | i.v. | 9 | I | UC-MSC | 0.5/1×10^7^ cells | >70% | ^13^ | |
| GVHD | N/A | 18~70 | i.v. | 32 | II | BMSC | 8/2×10^6^ cells/kg | ≥70% | ^14^ | |
| GVHD | N/A | 0.5~64 | i.v. | 55 | II | BMSC | 0.4×10^6^~9×10^6^/kg | >95% | ^15^ | |
| M/I | N/A | 36~66 | infarct-related artery, right antecubital vein | 9 | N/A | BMSC | 10^6^~10^8^ cells | 92%~96% | ^16^ | |
| Vocal Fold Scarring | NCT01981330 | ≥18 | injected into the vocal fold | 16 | I | MSC | 0.5×10^6^~1×10^6^ cells | >95% | ^17^ | |

i.v., intravenous injection; i.a., intra-articular knee injection; ADMSC, adipose derived mesenchymal stem cells; UC-MSC, umbilical cord mesenchymal stem cells; ALS, amyotrophic lateral sclerosis; ARDS, acute respiratory distress syndrome; M/I, myocardial infraction; GVHD, graft-versus-host disease; N/A, not available.

The clinical data were collected from U. S. National Library of Medicine (https://clinicaltrials.gov) and Pubmed (https://pubmed.ncbi.nlm.nih.gov).

**References**

1 Oh, K. W. et al. Repeated Intrathecal Mesenchymal Stem Cells for Amyotrophic Lateral Sclerosis. *Ann Neurol* **84**, 361-373, doi:10.1002/ana.25302 (2018).

2 Hare, J. M. et al. A randomized, double-blind, placebo-controlled, dose-escalation study of intravenous adult human mesenchymal stem cells (prochymal) after acute myocardial infarction. *J Am Coll Cardiol* **54**, 2277-2286, doi:10.1016/j.jacc.2009.06.055 (2009).

3 Wilson, J. G. et al. Mesenchymal stem (stromal) cells for treatment of ARDS: a phase 1 clinical trial. *Lancet Respir Med* **3**, 24-32, doi:10.1016/S2213-2600(14)70291-7 (2015).

4 Uccelli, A. et al. MEsenchymal StEm cells for Multiple Sclerosis (MESEMS): a randomized, double blind, cross-over phase I/II clinical trial with autologous mesenchymal stem cells for the therapy of multiple sclerosis. *Trials* **20**, 263, doi:10.1186/s13063-019-3346-z (2019).

5 Suk, K. T. et al. Transplantation with autologous bone marrow-derived mesenchymal stem cells for alcoholic cirrhosis: Phase 2 trial. *Hepatology* **64**, 2185-2197, doi:10.1002/hep.28693 (2016).

6 Matas, J. et al. Umbilical Cord-Derived Mesenchymal Stromal Cells (MSCs) for Knee Osteoarthritis: Repeated MSC Dosing Is Superior to a Single MSC Dose and to Hyaluronic Acid in a Controlled Randomized Phase I/II Trial. *Stem Cells Transl Med* **8**, 215-224, doi:10.1002/sctm.18-0053 (2019).

7 Chahal, J. et al. Bone Marrow Mesenchymal Stromal Cell Treatment in Patients with Osteoarthritis Results in Overall Improvement in Pain and Symptoms and Reduces Synovial Inflammation. *Stem Cells Transl Med* **8**, 746-757, doi:10.1002/sctm.18-0183 (2019).

8 Goto, T. et al. Phase I clinical trial of intra-bone marrow cotransplantation of mesenchymal stem cells in cord blood transplantation. *Stem Cells Transl Med*, doi:10.1002/sctm.20-0381 (2020).

9 Matthay, M. A. et al. Treatment with allogeneic mesenchymal stromal cells for moderate to severe acute respiratory distress syndrome (START study): a randomised phase 2a safety trial. *Lancet Respir Med* **7**, 154-162, doi:10.1016/S2213-2600(18)30418-1 (2019).

10 Bartolucci, J. et al. Safety and Efficacy of the Intravenous Infusion of Umbilical Cord Mesenchymal Stem Cells in Patients With Heart Failure: A Phase 1/2 Randomized Controlled Trial (RIMECARD Trial [Randomized Clinical Trial of Intravenous Infusion Umbilical Cord Mesenchymal Stem Cells on Cardiopathy]). *Circ Res* **121**, 1192-1204, doi:10.1161/CIRCRESAHA.117.310712 (2017).

11 Rodas, G. et al. Autologous bone marrow expanded mesenchymal stem cells in patellar tendinopathy: protocol for a phase I/II, single-centre, randomized with active control PRP, double-blinded clinical trial. *J Orthop Surg Res* **14**, 441, doi:10.1186/s13018-019-1477-2 (2019).

12 Lee, W. S., Kim, H. J., Kim, K. I., Kim, G. B. & Jin, W. Intra-Articular Injection of Autologous Adipose Tissue-Derived Mesenchymal Stem Cells for the Treatment of Knee Osteoarthritis: A Phase IIb, Randomized, Placebo-Controlled Clinical Trial. *Stem Cells Transl Med* **8**, 504-511, doi:10.1002/sctm.18-0122 (2019).

13 Ahn, S. Y., Chang, Y. S., Sung, S. I. & Park, W. S. Mesenchymal Stem Cells for Severe Intraventricular Hemorrhage in Preterm Infants: Phase I Dose-Escalation Clinical Trial. *Stem Cells Transl Med* **7**, 847-856, doi:10.1002/sctm.17-0219 (2018).

14 Kebriaei, P. et al. Adult human mesenchymal stem cells added to corticosteroid therapy for the treatment of acute graft-versus-host disease. *Biol Blood Marrow Transplant* **15**, 804-811, doi:10.1016/j.bbmt.2008.03.012 (2009).

15 Le Blanc, K. et al. Mesenchymal stem cells for treatment of steroid-resistant, severe, acute graft-versus-host disease: a phase II study. *Lancet* **371**, 1579-1586, doi:10.1016/S0140-6736(08)60690-X (2008).

16 Hofmann, M. et al. Monitoring of bone marrow cell homing into the infarcted human myocardium. *Circulation* **111**, 2198-2202, doi:10.1161/01.CIR.0000163546.27639.AA (2005).

17 Hertegard, S., Nagubothu, S. R., Malmstrom, E. & LeBlanc, K. Treatment of vocal fold scarring with autologous bone marrow-derived human mesenchymal stromal cells-first phase I/II human clinical study. *Stem Cell Res Ther* **11**, 128, doi:10.1186/s13287-020-01632-8 (2020).

Table S2.

**Table S2 The sequence of primers.**

| Name | Forward (5’-3’) | Reversed (5’-3’) |
| --- | --- | --- |
| iNOS | GGCAGCCTGTGAGACCTTTG | CATTGGAAGTGAAGCGTTTCG |
| CD206 | CTCTGTTCAGCTATTGGACGC | TGGCACTCCCAAACATAATTTGA |
| MerTK | AGCCTGAGGACTGCTTGGAT | CCTGGTGTGCAAGAGGCAAT |
| β-actin | GGCTGTATTCCCCTCCATCG | CCAGGTAACAATGCCATGT |
